# A New Method for Refining the GNSS-Derived Precipitable Water Vapor Map

**DOI:** 10.3390/s19030698

**Published:** 2019-02-08

**Authors:** Chen Liu, Nanshan Zheng, Kefei Zhang, Junyu Liu

**Affiliations:** 1 Jiangsu Key Laboratory of Resources and Environmental Information Engineering, China University of Mining and Technology, Xuzhou 221116, China; lcddhr@cumt.edu.cn; 2 School of Environment Science and Spatial Informatics, China University of Mining and Technology, Xuzhou 221116, China; kefei.zhang@rmit.edu.au; 3 SPACE Research Centre, School of Science, RMIT University, Melbourne VIC 3001, Australia; 4 College of Engineering, Northeastern University, Boston, MA 02115, USA; liujunyumail@gmail.com

**Keywords:** GNSS remote sensing, precipitable water vapor, weighted mean temperature, digital elevation model

## Abstract

The objective of the study was to put forth an interpolation method (the LZ method) for refining the GNSS-derived precipitable water vapor (PWV) map. We established a regional weighted mean temperature (*T_m_*) model for this experiment, which introduced a minor difference into the resultant GNSS-derived PWV compared to the previous *T_m_* models. The kernel of the LZ method consists of increasing the sample density via the virtual sample points. These virtual sample points originated from the digital elevation model (DEM) were constructed on the basis of the statistically significant correlation between PWV and geographical location (i.e., geographical coordinates and elevation). The LZ method was validated and compared to the conventional interpolation approach only accounting for the original sample points. The results reflect that the PWV maps generated by the LZ method showed more details than through conventional one. In addition, the prediction performance of the LZ method was better than that of the conventional method by using cross-validation.

## 1. Introduction

Water vapor is a key greenhouse gas and an indispensable component of the water cycle. Although it accounts for only 0.1% to 3% of the atmosphere, it is one of the most active atmospheric components [1]. It directly affects the vertical stability of the atmosphere and the formation or evolution of weather systems and contributes to radiation balance and a series of weather phenomena, such as cloud formation, rainfall, or snowfall events, by absorbing or releasing massive amounts of latent heat during phase transition [2]. Water vapor exhibits a complicated spatio-temporal distribution [3]. The accurate detection of the distribution and variation of atmospheric water vapor content can provide the data necessary to understand weather processes for weather forecasting and meteorology research [3]. Precipitable water vapor (PWV), the water vapor content of a vertically integrated column per unit area, is a direct indicator of atmospheric water vapor content and is expressed as the height of the corresponding equivalent liquid water column in centimeters [4].

The meteorological application of GNSS in the remote sensing of atmospheric water vapor has been a research hotspot since the early 1990s given the rapid development of GNSS and the extensive construction of continuous operation reference stations (CORS). The principle of the GNSS-based PWV retrieval technique was initially proposed by Bevis et al. [3] in 1992. This technique assumes that the wet component of atmospheric delays is proportional to PWV. It has attracted considerable attention since it was first reported because it can provide abundant data with high spatio-temporal resolution and is suitable for all weather conditions [5]. Many scholars have compared this method with traditional methods to verify its reliability. Differences among PWV data for North America [6], Europe [7,8,9], and Asia [10,11,12] retrieved from GNSS, radiosonde, and WVR observations were less than 5 mm. This result indicates that the accuracy of GNSS-derived PWV is comparable with that of traditional techniques.

In recent years, many studies [13] have concentrated on estimates of weighted mean temperature (*T_m_*), which is a key parameter in the conversion of zenith wet delay to PWV in GNSS meteorology. Actually, re-analysis products can provide sufficiently accurate *T_m_* data theoretically. However, the products have a problem with a time-delay release, cannot meet the real-time demand for *T_m_* data [13]. Therefore, *T_m_* model has been an indispensable part of GNSS meteorology due to it can be used to calculate *T_m_* values in real time. The current *T_m_* model is mainly divided into two categories, according to its differences in modeling principles. The first category can be called global model, usually modeled on the basis of *T_m_* spatiotemporal variations. These models with complex input parameters such as the geographic coordinates of the points to be computed and UTC time, but they can calculate the accurate *T_m_* values [14,15]. However, when determining *T_m_* from the global models, no meteorological data are needed and only the aforementioned parameters are required as input for these models. This reveals that the global models are very useful for those stations without surface meteorological sensors. The second category is the regional model, which is modeled on the basis of the relationship between *T_m_* and surface meteorological elements, is a linear model such as Bevis model [3] and Liu model [16]. Besides, the previous studies pointed out that the relationship between *T_m_* and surface meteorological elements is not constant, instead, it varies with location and time [17,18]. This indicates that the empirical regional model based on local meteorological data will be more accurate for the regional application. Therefore, in this work, we developed a multifactorial regional *T_m_* model using the datasets of radiosonde and surface meteorological data in Hong Kong, which was to meet the needs of the process of obtaining GNSS-derived PWV in Hong Kong.

The latest studies (e.g., [19,20]) have shown that GNSS-derived PWV has considerable potential applications in precipitation forecasting or meteorological disaster warning. Furthermore, the analysis of water vapor changing trends or the forecasting of short-term precipitation events requires the transformation of one-dimensional PWV data from observation networks to two-dimensional data through spatial interpolation [21]. The spatial interpolation algorithm consists of showing the dynamic distribution for water vapor within the CORS coverage area by real-time processing the GNSS-derived PWV of each receiver in the CORS network. Li et al. [20] developed a real-time monitoring and analytical system for the dynamic spatial and temporal variation characteristics of water vapor. Their system can track the dynamic variation in water vapor content and forecast small- and medium-scale extreme weather. However, there is limits the explanation of the PWV interpolation method and interpolation accuracy. In fact, few studies have focused on the reliability of the interpolation methods for PWV data. But, quite a few studies have confirmed that the Kriging interpolation method has good performance for meteorological variables, such as temperature [22], rainfall [23], and wind [24]. Meanwhile, high-density sample points with attributes like PWV as the input of Kriging interpolation would contribute to generating a more accurate continuous surface [25,26]. Realini et al. [27] found that even the densest GNSS networks experienced difficulties in providing data with the high spatial resolution for the detection of local fluctuations in water vapor. Because of economic reasons, it is unfeasible to further improve the density of GNSS networks. Hence this paper proposed an alternative method for increasing the sample density with inexpensive digital elevation model (DEM), which does not require equipment installation and maintenance costs. The DEM points with the three-dimensional coordinates were used to construct more sample points (i.e., virtual sample points) by multifactorial fitting equations. Moreover, the parameters of the multifactorial fitting equations are obtained in terms of the strong correlation between GNSS-derived PWV and the geographic coordinates as well as elevations of the original sample observations. In this work, the GNSS-derived PWV from the Hong Kong region was interpolated through two schemes: Scheme I only applying the PWV data derived from original stations in Hong Kong GNSS network; and Scheme II that combines the PWV data in Scheme I with that in the virtual observations. Prediction performances of the two schemes are evaluated using cross validation. Furthermore, the rationality of the GNSS-derived PWV maps obtained by the two schemes is assessed by the DEM maps and cumulative precipitation maps in the study area.

This paper is organized as follows: the study area, permanent GNSS network, and radiosonde station are described in Section 2. Methods for retrieval of precipitable water vapor, acquisition of a regional *T_m_* model, DEM point sampling, the establishment of virtual sample points, and spatial interpolation of PWV are discussed in Section 3. Section 4 presents test results of the proposed regional *T_m_* model and GNSS-derived PWV spatial interpolation. A summary and discussion are given in Section 5.

## 2. Study Area

**Figure 1:**
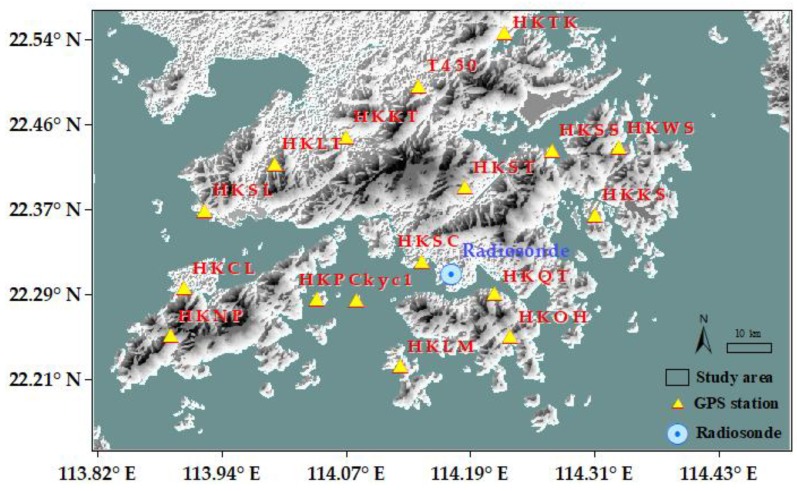
Map of the study area showing the satellite positioning reference stations in HK.

Hong Kong (HK) is situated in South China. It is located east of the Pearl River Estuary along the South China Sea. It has a marine subtropical monsoon climate with four distinctive seasons, and natural hillsides account for approximately 60% of the land area of HK (Figure 1). 

### 2.1. GNSS Network in HK

The HK Satellite Positioning Reference Station Network (SatRef) consists of 18 CORS, including 16 reference stations and two integrated monitoring stations. These stations constantly receive GNSS data. Observations and meteorological data from these stations are freely downloadable from the HK Survey & Mapping Office (https://www.geodetic.gov.hk/smo/index.htm) in RINEX format. These data can be used for high-precision positioning work and as fundamental data for retrieval algorithms in the remote sensing of tropospheric water vapor content. Water vapor products with high temporal and spatial resolution can be obtained given the continuous operation of the network and observation stations throughout HK. In this work, 17 out of 18 stations were selected for analysis and comparison given the continuity of observational and meteorological data. The details of these sites, including station name, geodetic coordinates, and elevations, are shown in Table 1.

### 2.2. Radiosonde Station in HK

A radiosonde station managed by the Hong Kong Observatory (HKO) is located in King’s Park and it launches radiosonde balloons at UTC 0:00 and 12:00 (local time UTC+8) daily. These radiosonde balloons can rise to approximately 30,000 m heights and record more than 100 layers of data, in the absence of extreme weather interference. The data include pressure, balloon height, temperature, and relative humidity. 

PWVs obtained from the meteorological data are also provided and the results have a high-accuracy [8]. However, given that the radiosonde balloons are only launched twice a day, meaning a 12-hr temporal resolution of the PWVs, which is too low for the monitoring of dynamic change in water vapor. For improving the accuracy of PWVs, based on a large amount of meteorological observation data in the HK region, a regional *T_m_* model can be developed.

The station number of King’s Park is 45004, and the sounding data of the station from 1973 to present could be acquired from the Wyoming Weather Website (http://weather.uwyo.edu/upperair/ seasia.html). The details of the station are shown in Table 1.

## 3. Materials and Methods

**Figure 2:**
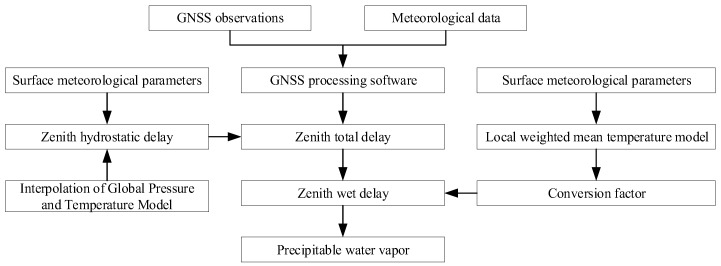
Flow chart for obtaining GNSS-derived PWV.

The PWV retrieval processing flow is shown in Figure 2. The software obtains the zenith total delay (ZTD) after processing GNSS observations and meteorological data. The empirical model is used to calculate the zenith hydrostatic delay (ZHD) on the basis of the measured or interpolated meteorological parameters. The conversion coefficient II is obtained by inputting the surface meteorological parameters in the regional model for the estimation of the weighted mean temperature (*T_m_*), which is used for a series of calculations. Then, the PWV is calculated by multiplying conversion coefficient II with zenith wet delay (ZWD). The details of the above steps are described in the following sections.

**Figure 3:**
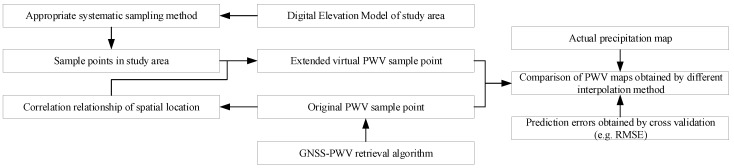
Flowchart for the comparison of the interpolation performances of the new method (based on extended virtual PWV sample points) and conventional one (based on original PWV sample points).

The proposed improved interpolation steps are shown in Figure 3. A series of points with three-dimensional coordinates, which are selected through the systematic sampling approach from the point cloud data of the DEM, establish links with original sample points to yield the virtual points with PWV values on the basis of the correlation between PWV values and geographical location. The original sample point and the virtual sample are adopted in the spatial interpolation method applied to construct the GNSS-derived PWV map. Finally, the qualities (i.e., the rationality and prediction performance) of interpolation is evaluated by actual precipitation map and prediction error obtained by cross-validation. The following sections describe these processes in detail.

### 3.1. Method for Water Vapor Retrieval 

The ground-based GNSS meteorology system is based on the GNSS observation network. The remote sensing of tropospheric water vapor involves the process of separating ZHD from ZTD and multiplying the obtained ZWD with conversion coefficient II to finally obtain the PWV value. These steps are illustrated by Equations (1)–(3):

(1) ZWD=ZTD−ZHD

(2) $$\Pi =\frac{10^{6}}{\rho_{w}\times R_{v}\times (\frac{k_{3}}{T_{m}}+k^{\prime }{}_{2})}$$

(3) PWV=Π×ZWD

where *ρ_w_* is the density of liquid water ($\rho_{w}=1.0g\cdot {\mathrm{cm}}^{-3}$) units not italic, and *R_v_* represents the universal gas constant for water vapor ($Rv=0.4613\;J\cdot g^{-1}\cdot K^{-1}$); $k^{\prime }{}_{2}$, *k*_3_ are atmospheric physical constants $k^{\prime }{}_{2}=22.1\pm 2.2\;K\cdot {\mathrm{hPa}}^{-1},k_{3}=(3.739\pm 0.012)\times 10^{5}{\;K}^{2}\cdot {\mathrm{hPa}}^{-1}$); *T_m_* indicates the weighted mean temperature in K [3]. 

ZHD in Equation (1) is calculated by using the Saastamoninen empirical model [28]:

(4) $$\mathrm{ZHD}=\frac{0.002277\times P}{1-0.00266\times \cos(2\varphi )-0.00028\times H}$$

where *P* is surface pressure (hPa). *ϕ* (rad) and *H* (km) represent the latitude and geodetic height of the station, respectively. *P* can be obtained from rinex meteorological data files that were released by the station. The value of *P* for stations that lack meteorological data can be calculated through interpolation with the global pressure and temperature model. Meanwhile, *ϕ* and *H* can be obtained from public receiver information.

ZTD in Equation (1) was estimated by using GAMIT software (version 10.6) [29] which is based on the double-difference model. The close distances between every two stations resulted in short baselines that subsequently strengthened the correlation between tropospheric parameters. To weaken this correlation, the observation data of four international GNSS service (IGS) stations (BJFS, SHAO, URUM, and LHAZ) were incorporated into the baseline solution [30,31]. In this work, and the remainder of the solution strategy for ZTD estimation is summarized in Table 2. 

Interval Zen/Number Zen = 1/25 shows that ZTD parameters are estimated per hour. In this study, the downloaded VMF1 model (ftp://everest.mit.edu//pub/GRIDS/) is adopted as the mapping function. 

### 3.2. Method for Obtaining T_m_

As shown in Equations (2) and (3), *T_m_* is a vital variable to determine the conversion in the process of ZWD to PWV [3], and it can be calculated with the following equation:

(5) $$T_{m}=\int (e/T)dz/\int (e/T^{2})dz$$

where *e* and *T* are the water vapor pressure and temperature of each layer of the atmosphere, respectively.

In fact, a continuous dataset (e.g., temperature) can hardly be acquired in practice, the numerical integration expressed by Equation (5) is often approximated as:

(6) $$T_{m}=\frac{\sum_{i=1}^{n}\left(\bar{e_{i}}/\bar{T_{i}}\right)\times \Delta h_{i}}{\sum_{i=1}^{n}\left(\bar{e_{i}}/{\bar{T_{i}}}^{2}\right)\times \Delta h_{i}}$$

where *h*_i_ is the thickness of *i*th layer atmosphere, and *e*_i_ and *Ti* are the water vapor pressure and temperature of the *i*th layer atmosphere, respectively.

A regional *T_m_* model for HK can be built on the basis of previous studies [16]. The relevant method and steps for developing a high accuracy *T_m_* model are described in detail in Section 4.

### 3.3. Method for DEM Point Sampling 

**Figure 4:**
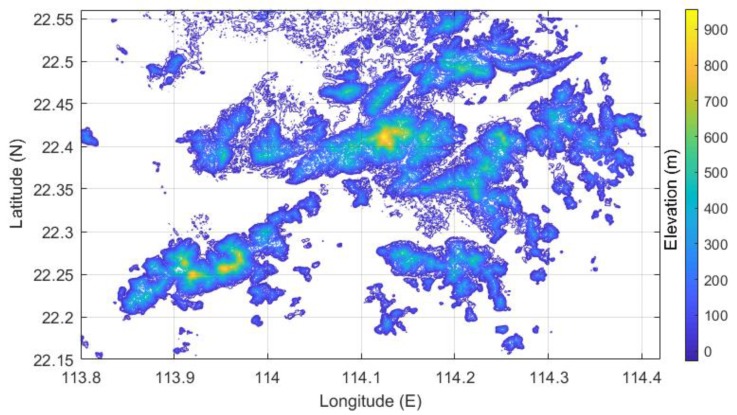
DEM of SRTM in HK area with a 3 arc-second resolution.

DEM is a continuous surface that represents ground elevation in the form of a series of ordered numerical arrays [32]. DEM data with a 3 arc-second resolution provided by the NASA Shuttle Radar Topographic Mission (SRTM, version 3) was adopted in this study. The vertical resolution of the DEM data was 90 m. The data used in this study are available on the website of the U.S. Geological Survey (https://dds.cr.usgs.gov/srtm/version2_1/SRTM3/). QGIS software (version: 2.8) [33] was used to clip out a rectangular area wherein HK was located at 113.80°E–114.42°E and 22.15°N–22.56°N. Then, the software was used to transform the clipped data into the xyz grid data format, which is a three-column matrix that comprises original geographic coordinates of DEM data. In this case, x, y, and z represent longitude, latitude, and ellipsoidal height, respectively. Next, these data were reorganized for display, and the SRTM DEM map of the study area is shown in Figure 4.

Massive amounts of DEM points result in reducing the efficiency of the interpolation algorithm. However, a series of points with spatial distribution determined by systematic sampling can be sufficient to illustrate the spatial variability of the research object. Thus, the approach only needs to extract a certain number of points by setting the appropriate longitude and latitude intervals. Besides, only the PWV data in the land surface needs to be generated in view of actual interpolation requirements. In this work, DEM samples were filtered on the basis of the following principles:

(1) Setting the longitude interval to 0.045°, a series of points were determined.
(2) The latitude interval was set to 0.035°, and all points within the scope of the study were determined.
(3) All points in the land area were retained and some points from the oceans were removed on the basis of the points identified above.

**Figure 5:**
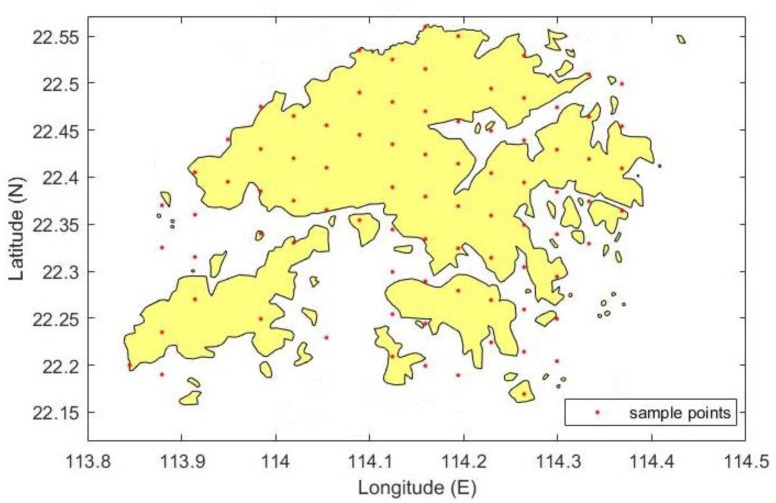
Distribution of 98 sample points selected through filtering approach.

After the above three steps, 98 points with three-dimensional coordinates were specified, as shown in Figure 5.

### 3.4. Method for the Establishment of Virtual Sample Points 

**Figure 6:**
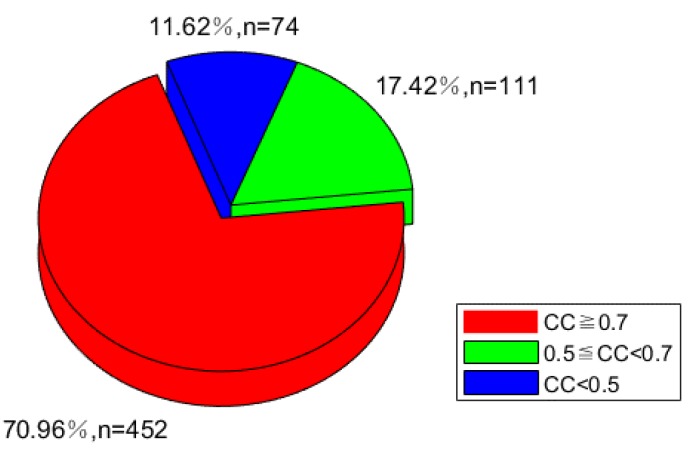
Pie chart of the CCs between PWV and ellipsoidal height of original points.

Only 17 stations have PWV information (as shown in Figure 1) that can be interpolated for the construction of water vapor distribution maps for HK. Moreover, the spatial distribution of these stations was insufficiently dense and uniform and was too coarse to reflect the dynamic fluctuations of water vapor. Thus, PWV information from existing original sample points to virtual sample points with a uniform distribution (Figure 5) must be extended through a reasonable method on the basis of the relationship between PWV values and the geographical position of 17 original points. The PWV values calculated with the GNSS observation data and meteorological data of 17 stations for the period of 0:00 on August 19, 2017 to 0:00 on September 1, 2017 (the sample interval was half an hour and provided 637 results in total) were compared with their elevation information (referred to ellipsoidal height in this paper) to explore the correlation between PWV and elevation. Figure 6 shows the number and percentage of points in the range of correlation coefficients (CCs). The CCs of 70.96% of the sample points exceeded 0.7, those of the remaining 17.42% were in the range of 0.5 to 0.7, and those of 11.62% of the sample points were less than 0.5. These results indicate that the PWV values and station elevation of more than 88% samples were significantly correlated. Therefore, extending PWV information to virtual sample points based on the correlation between station elevation (or horizontal position information) and PWV was reliable. The steps involved in extending PWV information are as follows:

(1) The CCs between station horizontal position (*x*, *y*, *x*^2^, *y*^2^, and *xy*)/elevation (*h* and *h*^2^) information and its PWV value were determined. Where *x*, *y*, and *h* represent longitude, latitude, and elevation, respectively.
(2) Station position parameters (e.g. *x*, *y*, and *h*) with CCs of less than 0.7 were deleted.
(3) The PWV expanding functional model based on the linear or nonlinear relations between PWV value and station spatial position information was constructed, and parameters were screened through the stepwise regression method:
  (7) PWV=A×B+C
  (8) $$A=\left[\begin{array}{l} a\\ b\\ c\\ d\\ e\\ f\\ g \end{array}\right],B=\left[\begin{array}{l} x\\ x^{2}\\ y\\ y^{2}\\ xy\\ h\\ h^{2} \end{array}\right],C=\left[\Delta_{x}+\Delta_{x^{2}}+\Delta_{y}+\Delta_{y^{2}}+\Delta_{xy}+\Delta_{h}+\Delta_{h^{2}}\right]$$
  where ${\left[a,b,c,d,e,f,g\right]}^{\prime }$ and $\left[\Delta_{x}+\Delta_{x^{2}}+\Delta_{y}+\Delta_{y^{2}}+\Delta_{xy}+\Delta_{h}+\Delta_{h^{2}}\right]$ are coefficient column vectors and constant row vectors of variables $\left[x,x^{2},y,y^{2},xy,h,h^{2}\right]$, respectively. Eventually, the optimal multiple regression equation is deduced by the stepwise method at the significance level of 0.05.

### 3.5. Method for Spatial Interpolation

Spatial interpolation is a common method used to obtain the information at a position within an unmeasured area and is based on the application of known information from surrounding stations [34], which are known as sample points. An interpolated value is also called predicted value. Some techniques, such as inverse distance weighted interpolation, Kriging, natural neighbor, and two-dimensional minimum curvature spline, are often used in the spatial interpolation. The Kriging technique is a geostatistical (rather than nondeterministic) approach that generates a continuous surface that does not pass through all sample points. The prediction provided by the Kriging technique is an unbiased estimate of the true value with the minimum variance. It has been used in a wide range of fields for years, including ecology, hydrology, meteorology, and geomatics [35,36,37,38]. Based on the existing research results [34,35,36,37,38] and the high correlation between PWV and elevation, in this study the co-Kriging (CK) method is adopted for spatial interpolation of PWV. In the case of CK, the predictions for points is defined as the following linear weighted model:

(9) $$Z(s_{0})=\sum_{i=1}^{n}\lambda_{i}z_{i}+\sum_{j=1}^{m}b_{j}x_{j}$$

where Z(*s*_0_) is the predicted value at the location *s*_0_; $z_{i},x_{j},i=1,\ldots ,n,j=1,\ldots ,m$ represent the values of the main variable and subvariable at locations *i* and *j*, respectively; *n* and *m* are the sample sizes of *z* and *x*, respectively; and *λ_i_* and *b_j_* are the CK weights, which depends on the spatial relationship between the values at the estimation point and the sample point. *λ_i_* and *b_j_* are obtained using the Lagrange multiplier as follows:

(10) $$\begin{array}{l} \sum_{i}^{n}\lambda_{i}\gamma \left(z_{i},z_{j}\right)+\sum_{i}^{m}b_{i}\gamma \left(x_{i},z_{j}\right)+\mu_{1}=\gamma \left(z_{0},z_{j}\right)\\ \sum_{i}^{n}\lambda_{i}\gamma \left(z_{i},x_{j}\right)+\sum_{i}^{m}b_{i}\gamma \left(x_{i},x_{j}\right)+\mu_{2}=\gamma \left(z_{0},x_{j}\right)\\ \sum_{i}^{n}\lambda_{i}=1\\ \sum_{i}^{n}b_{i}=0 \end{array}$$

where $\gamma \left(z_{i},z_{j}\right)$ is the value of the variogram between $s_{i}$ and $s_{j}$. Similarly, $\gamma \left(z_{0},z_{j}\right)$ is the value of the variogram between $s_{0}$ and $s_{j}$, et al. The dissimilarity between two sample points can be measured using a variogram, which is a function of the distance and direction of the two points. The values of $\left\{\lambda_{i}\right\}$ and $\left\{b_{j}\right\}$ are obtained by solving linear Equation (10) and then substituted into Equation (10) to interpolate CK at each point.

The prediction accuracy can be validated using the differences between the predicted values and the measured values at those sample points, because the latter can be assumed as the truth. In addition, the prediction performance of Kriging interpolation was evaluated by ‘one out’ cross-validation. The idea consists of removing temporarily one datum at a time from the data set and ‘re-predict’ this value on the basis of remaining data. Hence the predicted value at each point used to assess the Kriging interpolation performances comes from the ‘re-predict’. In this study, the statistical quantities used to evaluate the quality of a set of interpolation results follow:

(1) Mean error (ME) is the averaged difference between the predicted and measured values. Values close to 0 are preferred. The equation for ME is as follows:
  (11) $$\mathrm{ME}=\frac{1}{n}\sum_{i=1}^{n}\left(\hat{Z}(s_{i})-z(s_{i})\right)$$
  where $\hat{Z}(s_{i})$ and $z(s_{i})$ are the predicted and measured values at location *S_i_*, respectively, and *n* is the number of the sample points.
(2) Root mean square error (RMSE) is also the deviation between the predicted and measured values. Small RMSE values indicate improved accuracy. This index is calculated as follows:
  (12) $$\mathrm{RMSE}=\sqrt{\frac{1}{n}\sum_{i=1}^{n}{\left(\hat{Z}(s_{i})-z(s_{i})\right)}^{2}}$$
(3) Root mean square standardized error (RMSSE) can be also used to evaluate the quality of a set of prediction. The value of this factor should be close to 1 if the prediction standard errors are valid. Values close to 1 are indicative of good prediction accuracy. Equation (13) shows the equation of this factor:
  (13) $$\mathrm{RMSSE}=\sqrt{\frac{1}{n}\sum_{i=1}^{n}\left[{\left(\hat{Z}(s_{i})-z(s_{i})\right)}^{2}/\sigma^{2}\left(s_{i}\right)\right]}$$
  where $\sigma^{2}\left(s_{i}\right)$ is the variance of the prediction at location *S_i_*.

## 4. Experiments and Results 

### 4.1. Modeling of the Regional T_m_ for HK

In general, the values of *T_m_* computed by Equation (6) are only taken as the truth for validating. Besides this the *T*_m_ usually obtained by the regional *T_m_* model based on surface meteorological elements such as surface temperature (*T_s_*). Such model was first proposed by Bevis et al. [3] in 1992, which was the linear regression model between *T_m_* and *T_s_* ($T_{m}=70.2+0.72T_{s}$) with an RMSE of 4.74 K. In recent years, many further studies [39] have introduced all the meteorological elements (i.e., *T_s_*, pressure (*P*), and water vapor pressure (e)) obtained by the meteorological sensors to build multivariate regression equations to further improve the accuracy of the regional models. However, the effect of this approach is not ideal. In this paper, 1454 datasets, including *T_m_*, *T_s_*, *P*, and *e*, acquired during the period of January 1, 2015 to December 31, 2016, were used for modeling the regional *T_m_* model. *T_m_* results were calculated by the numerical integration method through using daily sounding data. Other experimental data (*T_s_*, *P*, and *e*) were derived from the observations obtained by radiosonde stations.

Table 3 lists the CCs for the relationships between every two elements. It can be found the CCs between *T_m_* and *T_s_*/*P*/*e* were 0.862/0.833/−0.744, respectively. The result reveals that the independent variable *T_s_* exerted the most intense effect on the dependent variable *T_m_*. This relationship validates the rationality of the traditional single-factor model that takes *T_s_* as the unique argument. At the same time, the CCs between each variable exceeded 0.7. The findings indicate that the arguments of the *T_m_* regression model were not mutually independent and were instead strongly correlated. Hence adding all meteorological elements into the regression model failed to improve the model prediction accuracy.

JASP software (version 0.9) [40] was adopted to weaken the influence of multiple collinearities through the stepwise regression method. A total of 1,454 sets of values of *T_s_*, *P*, and *e* and their corresponding *T_m_* values were used to establish the regression model for the experimental period. Stepwise regression analysis yielded the following two models that passed the significance test at the 95% confidence level:

(14) $$\begin{array}{l} 1:T_{m}=0.603T_{s}+107.632\\ 2:T_{m}=0.447T_{s}+0.117e+150.787 \end{array}$$

Equation (14) shows that Model 1 contains the unique independent variable *T_s_*, (K) and the independent variable of Model 2 includes *e* (hPa) in addition to *T_s_*.

### 4.2. Analysis of the Proposed T_m_ Model

*R*, *R^2^*, and S.E. values derived from regression models are presented in Table 4 and used to validate the fit of the proposed models. Here, *R, R^2^*, and S.E. represent the standard regression coefficient, the coefficient of determination, and the standardized error of the estimate, respectively. Model 2 had higher *R* and *R^2^* values and smaller S.E. values than Model 1. Thus, the fitting precision of Model 2 is higher than that of Model 1. We choose model 2 that is a multifactorial regional model (named the MR model in this study) with the better fit to the test data as the proposed new model for the further test.

Although the MR model fitted the test data well, the prediction ability of the MR model maybe not better than that of the widely used Bevis model and previous HK regional model (or called Liu model $T_{m}=85.63+0.668T_{s}$ [16,37]). Therefore, assessing the prediction capacity of the proposed model using recent data is necessary. In this experiment, 180-day radiosonde data for the period from DOY 1 to 180, 2017 were selected to compute the true value of *T_m_* through the numerical integration method. The calculation results of the MR model, Liu model, and Bevis model were compared by using meteorological data at the corresponding sampling time epochs (00Z and 12Z during the experimental period). 

**Figure 7:**
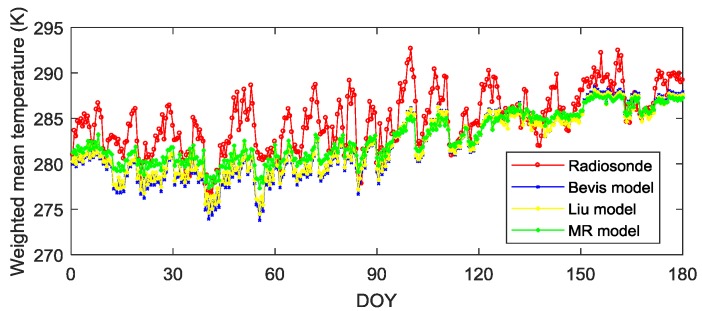
Comparison of the *T_m_* time series obtained from the numerical integration method (by using radiosonde data), the MR model, Liu model, and Bevis model for the period from DOY 1 to DOY 180, 2017. The radiosonde station acquired data at 00Z and 12Z UTC on each day.

Figure 7 presents the comparison of various models and truth values. Obviously, the pink line (the MR model) was more consistent with the red line (radiosonde) than both with the blue line (Bevis model) and yellow line (Liu model). These results indicate that the calculations provided by the MR model better match the truth value. Moreover, RMSE statistics between the prediction values of the three models and true values are listed in Table 5. It can be seen the RMSE of the MR model was lower than that of both Bevis and Liu empirical model. Specifically, the RMSE value of the MR model decreased by 0.7 K from 3.5 K to 2.8 K compared to Bevis model, which reduced 0.5 K from 3.3 K to 2.8 K than that of Liu model. Thus, the *T_m_* prediction accuracy of the MR model had been improved by 20% and 15% compared to the Bevis model and Liu model, respectively. Besides, the *T_m_* calculated using three aforementioned models had errors, and it is necessary to assess the impact of the error on the accuracy of the resultant PWV. The relative error in the PWV is a commonly used quantity to evaluate the impact of the error in *T_m_* on its resultant PWV, which is computed by:

(15) $$\frac{\Delta \mathrm{PWV}}{\mathrm{PWV}}\approx \frac{T_{m}+\Delta T_{m}}{T_{m}}-1=\frac{\Delta T_{m}}{T_{m}}$$

where ΔPWV is the error in PWV resulting from the error in *T_m_* and Δ*T_m_*. This is a simplified equation for calculating the relative error in the PWV. Interested readers can refer to [18] for a detail derivation process of the equation. Equation (15) reveals that relative error of *T_m_* can approximate the relative error of PWV. Furthermore, the relative RMSE (named ΔRMSE) of PWV can be calculated using the equation as follows [18]:

(16) $$\Delta \mathrm{RMSE}=\frac{\mathrm{RMSE}}{T_{m}}$$

As shown in Table 5, the ΔRMSE of the MR model (0.9%) is smaller than that of both Bevis model (1.2%) and Liu model (1.1%). This result shows that the MR model introduced a lower relative error into the resultant PWV compared with the Bevis model and Liu model. Therefore, the MR model with high prediction accuracy was applied in the following PWV interpolation experiments.

### 4.3. PWV Spatial Interpolation and Precipitation Distribution Maps

Under the influence of typhoon Hato (International number: 1713), a large area in HK received heavy rainfall from August 22, 2017 to August 23, 2017. In this work, the GNSS observation and meteorological data of 17 stations at the corresponding time of DOY 234 to 235, 2017 were selected. Meanwhile, the precipitation data were collected from HKO as the auxiliary information for validation. The interpolation experiments were implemented in two Schemes, Scheme I, which involves interpolation using CK method based on the original 17 measured points in SatRef, and Scheme II, which is the proposed interpolation method (named LZ method in this study) that combines17 measured points with 98 virtual measured points to interpolate by CK. 

**Figure 8:**
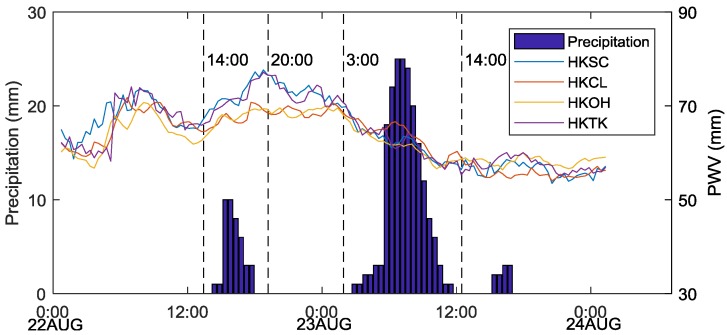
Relationship between the PWV (line) of four selected stations and average precipitation in HK (histogram) for the period during 22–24 August 2017.

PWV information retrieved from four selected stations, namely, HKSC, HKCL, HKOH, and HKTK and the half-hour total precipitation for HK from August 22 to 24, 2017 are shown in Figure 8. The PWV peaked after a certain period of accumulation, and precipitation occurred after the PWV had decreased for several hours. The PWV value decreased after an intense precipitation event. Therefore, there is a certain relationship between PWV and actual precipitation. 

**Figure 9:**
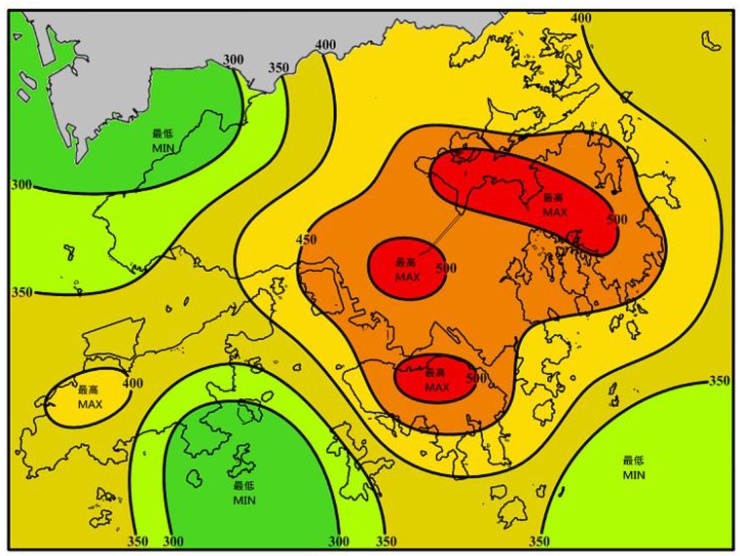
Precipitation map for August 2017 (isohyets are in mm).

**Figure 10:**
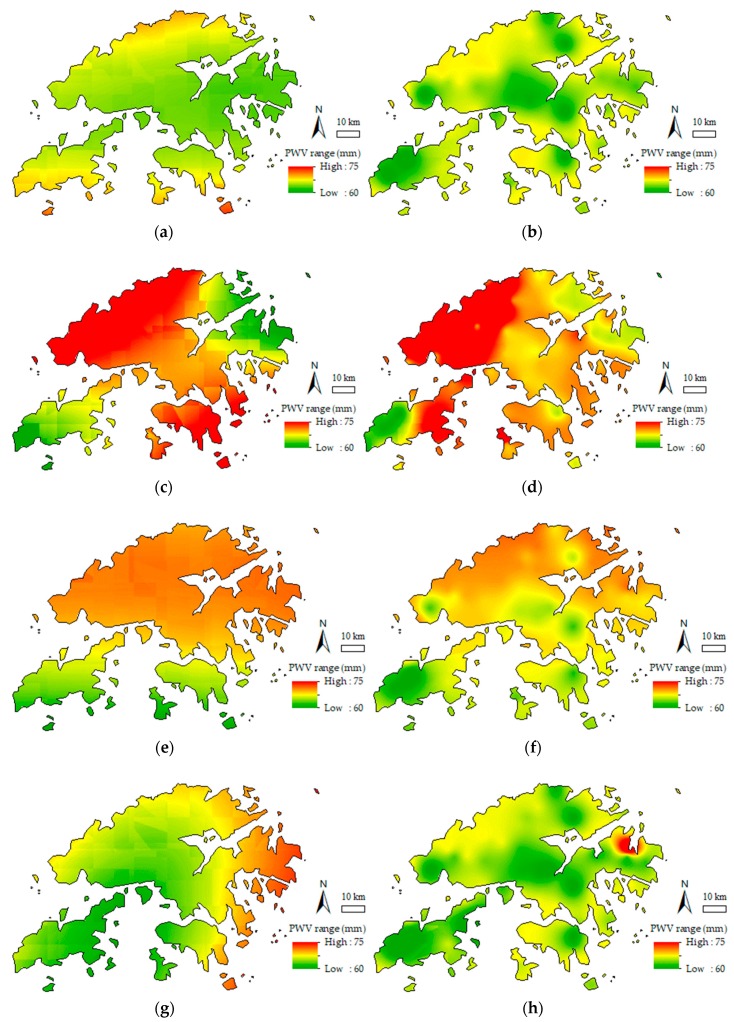
CK interpolation results for PWV provided by Schemes I and II. Graph (**a**,**c**,**e**), and (**g**) are generated by Scheme I, while graph (**b**,**d**,**f**), and (**h**) are generated by Scheme II.

**Figure 11:**
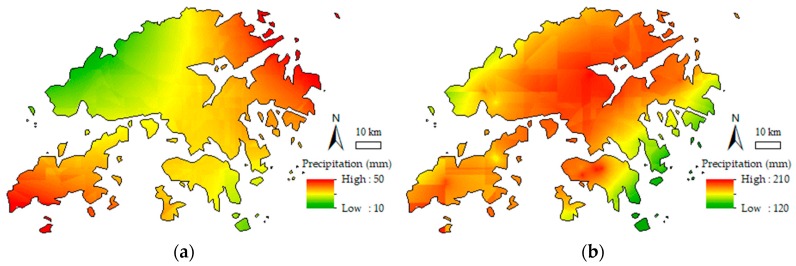
Precipitation distribution during the selected period. (**a**) Accumulated precipitation at 14:00 to 20:00 on August 22, 2017; (**b**) Accumulated precipitation at 03:00 to 14:00 23rd August 2017.

As shown in Figure 8, the data for four specific time epochs, including 12:00 and 20:00 on August 22, 2017 and 3:00 and 14:00 on August 23, 2017 were subjected to PWV CK interpolation experiments with Schemes I and II. Meanwhile, Figure 9 shows the August 2017 precipitation map released by the HKO. The GNSS-derived PWV distribution maps constructed by the two interpolation schemes are shown in Figure 10, and Figure 11 shows the cumulative precipitation for the corresponding time intervals. 

In Figure 10a–h, the PWV distribution maps generated by Schemes I and II indicate that the PWV in the central area was lower than that in the northern and northwestern fringe areas. Mea3nwhile, it can be found that the central area with lower PWV values than surrounding parts, however, its elevation is significantly higher than in others (recall Figure 4). This finding consistent with the previous research [41] that the PWV tends to decrease with increasing elevation. Similarly, the PWV in the central of the island in the southwest of HK is relatively lower than other parts in accordance with Figure 10b,d,f,h. On the contrary, the elevation of the central part is higher than the rest of the island. Nevertheless, the phenomenon, which the PWV values in the central region are lower than in the rest part of the island, shown in Figure 10b,d,f,h obtained from Scheme II is not easy to find in corresponding Figure 10a,c,e,g obtained from Scheme I. In consequence, Scheme I provided the general distribution map of PWV that was influenced by the sparsity of original sample points. In contrast, Scheme II, with many virtual sampling points provided the detailed spatial distribution map of PWV. Furthermore, the interpolation results of Scheme I (in Figure 10a,c,e,g) demonstrated the dynamic change of PWV during the period (August 22 to 24, 2017). The PWV in the study area was low at 14:00 on August 22, 2017 and reached than 70 mm at 20:00 on the same day. Besides, the PWV was lower in the central and southern but higher in the northern and northwestern at 3:00 on August 23, before heavy rainfall. The PWV in HK deeply deceased after heavy rainfall at 14:00 on August 23, 2017. Thus, the PWV in the central and southwestern was less than that in the northeastern. In addition, the PWV map generated by the LZ method (Scheme II) show a similar changing trend and it is significant it has more details of PWV distribution. 

For evaluating the performance, precipitation distribution maps generated by actual cumulative rainfall was compared with the interpolation results of the two schemes. Figure 11 shows the distribution of accumulated precipitation in the period from 14:00 to 20:00 on August 22, 2017 and between 03:00 and 14:00 on August 23, 2017. The total rainfall in the whole study area ranged from 14 mm to 46 mm during the period between 14:00 and 20:00 on August 22 and from 129 mm to 201 mm during 03:00 to 14:00 on August 23, 2017. Figure 11a shows increased precipitation in the southwestern islands and northeastern regions, and Figure 11b indicates that precipitation increased in the central mountainous area and southwestern islands. Besides, Figure 9 provides the rainfall map in August 2017 released by the HKO (isohyets are in mm), which shows that rainfall mainly localized in the central, northeastern, and southwestern area in the HK. Figure 9 and Figure 4 reveal that the area with higher elevation results in higher precipitation, and vice versa. In other words, there is a spatial auto-correlated effect within the precipitation and elevation in the HK. However, we also find that the area with heavy precipitation (the elevation is higher than the surrounding) has smaller PWV than the surrounding. The research of Goovaerts [42] may provide an explanation for the phenomenon. Water vapor (expressed as PWV) condenses due to adiabatic cooling caused by terrain uplifting, eventually forming precipitation. Therefore, the interpolation results of the two schemes presented in Figure 10 indicates that the PWV distribution interpolated by Scheme II is closer to the real than that interpolated by Scheme I.

### 4.4. Error Analysis of the Two Interpolation Schemes

The CK interpolation results of the two schemes for all selected time epochs were cross-validated in terms of ME, RMSE, and RMSSE to verify the superiority of the proposed method. This approach aims to verify the fitness of the continuous surfaces generated by two schemes to the 17 original points with PWV information. Thus, these accuracies performance indicators (ME, RMSE and RMSSE) reflect the differences between the PWV values estimated in two scheme and GNSS-derived PWV estimated by GAMIT at each original sample points, and the latter can be assumed as the truth.

The selected time epochs included 14:00 and 20:00 on August 22, 2017 and 03:00 and 14:00 on August 23, 2017 and were simplified as time epochs 1 to 4. As shown in Table 6, ME and RMSE values of Scheme II were lower than those of Scheme I, whereas the RMSSE of Scheme II was closer to 1 than that of Scheme I. The mean ME and RMSE of Scheme I decreased by 0.06 and 3.27 mm from 0.07/6.47 mm to 0.01/3.20 mm, respectively. The mean RMSSE value of Scheme II was 0.41 lower than that of Scheme I, which was closer to 1. Furthermore, the minimum ME of Scheme I (0.02 mm) and Scheme II (−0.01 mm) were obtained for time epoch 3. The minimum RMSE values of both of the two Schemes were 3.23 and 1.61 mm, respectively, and were obtained for time epoch 3. The RMSSE values for time epoch 1 were the closest to 1. Accordingly, the maximum ME and RMSE of Scheme I were 0.25 and 10.62 mm, respectively, and were obtained for time point epoch 4/2, respectively. The maximum ME and RMSE of Scheme II were 0.05 and 5.58 mm, respectively, and were obtained at time epoch 1/2. Moreover, the RMSSE values of Scheme I for time epoch 3 (1.61 mm) and Scheme II for time epoch 2 (0.97 mm) were farthest from 1. To summarize, the series of PWV prediction points calculated by using the proposed method (Scheme II) were in good agreement with original measurement points. Scheme II provided low ME and RMSE values and RMSSE values close to 1. These results indicate that the accuracy of Scheme II is higher accuracy than that of Scheme I. The most outstanding advantage of Scheme II is that the density of input points with PWV as the CK interpolation algorithm is much denser than that of Scheme I. When the CK interpolation algorithm is taken to predict value at each unknown point in the study area, there are many more known points of Scheme II than that of Scheme I. 

## 5. Summary and Discussion

In this research, we have proposed a new interpolation method (the LZ method) for refining the GNSS-derived PWV distribution map. In addition, a multifactorial regional *T_m_* model (the MR model) for the demand of the LZ method test experiment was proposed. The relative RMSE results reflect that compared with the previous *T_m_* model (i.e., Bevis model and Liu model), the MR model induced less difference into the resultant GNSS-derived PWV. The kernel of the LZ method consists of densifying the sample points by providing virtual sample points. Based on the statistically significant correlation within PWV and geographic coordinates/elevation at 17 original sample points, PWVs were extended from the original 17 stations to 98 uniformly distributed DEM virtual sampling points. Four-time epochs during the period from August 22 to 23, 2017 were selected to check the performance of the LZ method. The results indicate that the PWV maps generated by the LZ method have more fact-based details than that through the conventional interpolation method with only 17 original sampling points. Many more areas in the PWV map have a tendency that the value of PWV decreases with the increasing elevation. Moreover, the precipitation maps show that there is a positive correlation between precipitation and elevation in the HK. In additions, all of the accurate indicators (i.e., ME, RMSE, and RMSSE) show that the LZ method has better performance than the conventional method.

Overall, the LZ method on the basis of the application of virtual sample points resolved the insufficient horizontal resolution of PWV interpolation results caused by the sparse and uneven distribution of GNSS stations. Future work is to analyze the accuracy of the proposed approaches within different weather conditions or in different locations.

## Figures and Tables

**Table 1:** GNSS stations and radiosonde station in HK selected for experiment.

Station Name	Longitude E (°)	Latitude N (°)	Height (m)
**HKCL**	113.91	22.30	7.714
**HKKS**	114.31	22.37	44.718
**HKKT**	114.07	22.44	34.576
**HKLM**	114.12	22.22	8.554
**HKLT**	114.00	22.41	125.922
**HKNP**	113.89	22.25	350.672
**HKOH**	114.23	22.25	166.401
**HKPC**	114.04	22.28	18.130
**HKQT**	114.21	22.29	5.178
**HKSC**	114.14	22.32	20.239
**HKSL**	113.93	22.37	95.297
**HKSS**	114.27	22.43	38.714
**HKST**	114.18	22.40	258.705
**HKTK**	114.22	22.55	22.533
**HKWS**	114.34	22.43	63.791
**kyc1**	114.08	22.28	116.380
**T430**	114.13	22.49	41.323
**King’s Park (45004)**	114.17	22.31	66.0

**Table 2:** Solution strategy for obtaining ZTD.

Parameter	Strategy
Interval Zen/Number Zen	1/25
Elevation Cutoff	10°
Mapping Function	VMF1

**Table 3:** CCs of various meteorological elements.

CC	*T_m_*	*T_s_*	*e*	*P*
***T_m_***	1	0.862	0.833	−0.744
***T_s_***	0.862	1	0.926	−0.851
***e***	0.833	0.926	1	−0.843
***P***	−0.744	−0.851	−0.843	1

**Table 4:** Goodness-of-fit results from the two regression models.

Model	*R*	*R^2^*	S.E
1	0.862	0.743	1.932
2	0.867	0.752	1.901

**Table 5:** RMSE and ΔRMSE between *T_m_* results of the MR, Liu and Bevis models from radiosonde during test time (Unit: K).

Evaluation Indices	MR Model	Liu Model	Bevis Model
RMSE	2.8	3.3	3.5
ΔRMSE	0.9%	1.1%	1.2%

**Table 6:** Prediction accuracies of CK in terms of ME, RMSE, and RMSSE resulting from the two schemes and the selected time epoch (unit: mm).

Time Epoch	ME		RMSE		RMSSE	
	Scheme I	Scheme II	Scheme I	Scheme II	Scheme I	Scheme II
1	0.18	0.05	5.63	3.09	1.25	1.02
2	−0.18	−0.05	10.62	5.58	1.35	0.97
3	0.02	−0.01	3.23	1.61	1.61	1.02
4	0.25	0.02	6.37	2.54	1.47	1.02
Mean	0.07	0.01	6.47	3.20	1.42	1.01

## References

[1] Torres B., Cachorro V.E., Toledano C., Galisteo J.P.O.D., Berjón A., de Frutos A.M., Bennouna Y., Laulainen N. (2010). Precipitable water vapor characterization in the Gulf of Cadiz region (southwestern spain) based on Sun photometer, GPS, and radiosonde data. J. Geophys. Res..

[2] Mattar C., Sobrino J.A., Julien Y., Morales L. (2011). Trends in column integrated water vapour over Europe from 1973 to 2003. Int. J. Climatol..

[3] Bevis M., Businger S., Herring T.A., Rocken C., Anthes R.A., Ware R.H. (1992). GPS meteorology: Remote sensing of atmospheric water vapor using the global positioning system. J. Geophys. Res..

[4] Holloway C.E., Neelin J.D. (2010). Temporal relations of column water vapor and tropical precipitation. J. Atmos. Sci..

[5] Jin S., Park J.-U., Cho J.-H., Park P.-H. (2007). Seasonal variability of GPS-derived zenith tropospheric delay (1994–2006) and climate implications. J. Geophys. Res..

[6] Dai A., Wang J., Ware R.H., Van Hove T. (2002). Diurnal variation in water vapor over North America and its implications for sampling errors in radiosonde humidity. J. Geophys. Res..

[7] Gurbuz G., Jin S. (2017). Long-time variations of precipitable water vapour estimated from GPS, MODIS and radiosonde observations in Turkey. Int. J. Climatol..

[8] Li Z., Muller J.-P., Cross P. (2003). Comparison of precipitable water vapor derived from radiosonde, GPS, and Moderate-Resolution Imaging Spectroradiometer measurements. J. Geophys. Res..

[9] Van Baelen J., Aubagnac J.-P., Dabas A. (2005). Comparison of near–real time estimates of integrated water vapor derived with GPS, radiosondes, and microwave radiometer. J. Atmos. Ocean. Technol..

[10] Ohtani R., Naito I. (2000). Comparisons of GPS-derived precipitable water vapors with radiosonde observations in Japan. J. Geophys. Res..

[11] Liou Y.-A., Teng Y.-T., Van Hove T., Liljegren J.C. (2001). Comparison of precipitable water observations in the near tropics by GPS, microwave radiometer, and radiosondes. J. Appl. Meteorol..

[12] Kwon H.-T., Iwabuchi T., Lim G.-H. (2007). Comparison of precipitable water derived from ground-based GPS measurements with radiosonde observations over the Korean Peninsula. J. Meteorol. Soc. Jpn..

[13] Ding M. (2018). A neural network model for predicting weighted mean temperature. J. Geodesy..

[14] Yao Y., Xu C., Zhang B., Cao N. (2014). GTm-III: A new global empirical model for mapping zenith wet delays onto precipitable water vapour. Geophys. J. Int..

[15] Böhm J., Möller G., Schindelegger M., Pain G., Weber R. (2014). Development of an improved empirical model for slant delays in the troposphere (GPT2w). GPS Solut..

[16] Liu Y., Chen Y., Liu J. (2001). Determination of weighted mean tropospheric temperature using ground meteorological measurement. Geo-Spat. Inf. Sci..

[17] Wang J., Zhang L., Dai A. (2005). Global estimates of water-vapor-weighted mean temperature of the atmosphere for GPS applications. J. Geophys. Res..

[18] Wang X., Zhang K., Wu S., Fan S., Cheng Y. (2016). Water vapor-weighted mean temperature and its impact on the determination of precipitable water vapor and its linear trend. J. Geophys. Res. Atmos..

[19] Bordi I., Raziei T., Pereira L.S., Sutera A. (2015). Ground-based GPS measurements of precipitable water vapor and their usefulness for hydrological applications. Water Resour. Manag..

[20] Li L., Yuan Z., Luo P., Shen J., Long S., Zhang L., Jiang Z. A system developed for monitoring and analyzing dynamic changes of GNSS precipitable water vapor and its application. Proceedings of the China Satellite Navigation Conference.

[21] Chen B., Dai W., Liu Z., Wu L., Kuang C., Ao M. (2018). Constructing precipitable water vapor map from regional GNSS network observations without collocated meteorological data for weather forecasting. Atmos. Meas. Tech..

[22] Xuan T.N., Ba T.N., Khac P.D., Quang H.B., Thi N.T.N., Van Q.V., Thanh H.L. (2015). Spatial interpolation of meteorologic variables in vietnam using the kriging method. J. Inf. Proc Syst..

[23] Wardah T., Sharifah Nurul Huda S.Y., Deni S.M., Nur Azwa B. Radar rainfall estimates comparison with kriging interpolation of gauged rain. Proceedings of the 2011 IEEE Colloquium on Humanities, Science and Engineering.

[24] Berndt C., Haberlandt U. (2018). Spatial interpolation of climate variables in Northern Germany—Influence of temporal resolution and network density. J. Hydrol..

[25] Setiyoko A., Arymurthy A.M., Arief R. Effects of different sampling densities and pixel size on kriging interpolation for predicting elevation. Proceedings of the 2018 International Conference on Signals and Systems (ICSigSys).

[26] Bradley S.G., Dirks K.N., Stow C.D. (1998). High resolution studies of rainfall on Norfolk Island, Part III: A model for rainfall redistribution. J. Hydrol..

[27] Realini E., Sato K., Tsuda T., Oigawa M., Iwaki Y., Shoji Y., Seko H. (2015). Local-Scale precipitable water vapor retrieval from high-elevation slant tropospheric delays using a dense network of GNSS receivers. IAG 150 Years.

[28] Saastamoinen J. (1972). Contributions to the theory of atmospheric refraction. Bulletin Géodésique.

[29] Li G., Wang H. Using GAMIT to derive the precipitabel water vapor. Proceedings of the 2010 International Conference on Multimedia Technology.

[30] Wang Z., Xin P., Li R., Wang S. (2017). A Method to reduce non-nominal troposphere error. Sensors.

[31] Yang F., Guo J., Shi J., Zhou L., Xu Y., Chen M. (2018). A method to improve the distribution of observations in GNSS water vapor tomography. Sensors..

[32] Mukherjee S., Garg R.D., Raju P.L.N. (2013). Evaluation of topographic index in relation to terrain roughness and DEM grid spacing. J. Earth Syst. Sci..

[33] QGIS Software. https://www.qgis.org/en/site/.

[34] Meng Q., Liu Z., Borders B.E. (2013). Assessment of regression kriging for spatial interpolation—Comparisons of seven GIS interpolation methods. Cartogr. Geogr. Inf. Sci..

[35] Addink E.A. (1999). A comparison of conventional and geostatistical methods to replace clouded pixels in NOAA-AVHRR images. Int. J. Remote Sens..

[36] Ashiq M.W., Zhao C., Ni J., Akhtar M. (2009). GIS-based high-resolution spatial interpolation of precipitation in mountain–plain areas of Upper Pakistan for regional climate change impact studies. Theor. Appl. Climatol..

[37] Bostan P.A., Heuvelink G.B.M., Akyurek S.Z. (2012). Comparison of regression and kriging techniques for mapping the average annual precipitation of Turkey. Int. J. Appl. Earth Obs. Geoinf..

[38] Alvarez O., Guo Q., Klinger R.C., Li W., Doherty P. (2013). Comparison of elevation and remote sensing derived products as auxiliary data for climate surface interpolation. Int. J. Climatol..

[39] Wang X., Song L., Dai Z., Cao Y. (2011). Feature analysis of weighted mean temperature *T_m_* in Hong Kong. J. Nanjing. Univ. Inf. Sci. Technol. Nat. Sci. Edn..

[40] JASP Software. https://jasp-stats.org/.

[41] Onn F., Zebker H.A. (2006). Correction for interferometric synthetic aperture radar atmospheric phase artifacts using time series of zenith wet delay observations from a GPS network. J. Geophys Res..

[42] Goovaerts P. (2000). Geostatistical approaches for incorporating elevation into the spatial interpolation of rainfall. J. Hydrol..

